# Applications of transfer learning in sunflower disease detection: advances, challenges, and future directions

**DOI:** 10.55730/1300-0152.2763

**Published:** 2025-10-06

**Authors:** Yonis GULZAR

**Affiliations:** Department of Management Information Systems, College of Business Administration, King Faisal University, Al-Ahsa, Saudi Arabia

**Keywords:** Sunflower disease detection, transfer learning, convolutional neural networks, lightweight models, federated learning, explainable artificial intelligence

## Abstract

**Background/aim:**

Sunflower (*Helianthus annuus*) is a crop of high economic and nutritional importance that continues to suffer significant yield losses due to foliar diseases. Traditional image-based and laboratory detection techniques remain limited by subjectivity, cost, and scalability. Transfer learning (TL) has recently emerged as an effective approach to overcoming these challenges involving the reuse of pretrained deep models for plant pathology tasks. Presented here is a systematic examination of recent TL-based studies on sunflower disease classification to identify prevailing trends, research gaps, and future opportunities.

**Materials and methods:**

A structured Scopus query was employed to retrieve peer-reviewed articles published between 2021 and 2025. Strict inclusion and exclusion criteria ensured technical relevance to TL-based sunflower disease detection. Subsequently, 30 studies meeting the criteria were critically reviewed and analyzed in terms of model architecture, dataset characteristics, preprocessing strategies, and reported evaluation metrics. The comparative assessment focused on convolutional neural networks (CNNs), transformer-based architectures, and hybrid models.

**Results:**

The analysis revealed a dominant reliance on pretrained CNNs such as ResNet, VGG, Inception, and EfficientNet. Several studies employed lightweight or federated learning variants to enhance deployment feasibility under field conditions. Among the commonly observed challenges were limited dataset diversity, class imbalance, and insufficient explainability. A key word cooccurrence analysis indicated an evolving research focus, transitioning from basic deep learning implementation to explainable and privacy-preserving frameworks optimized for edge devices.

**Conclusion:**

The review revealed substantial progress in TL applications for the diagnosis of sunflower disease but underscored the need for larger, standardized datasets and cross-regional validation. Future studies should prioritize interpretable, adaptive architectures that can function in real-world agricultural environments. The insights drawn from this synthesis extend beyond sunflower pathology, offering a foundation for scalable, domain-transferable TL solutions in broader plant disease detection contexts.

## 1. Introduction

### 1.1. Background and motivation

**Figure 1:**
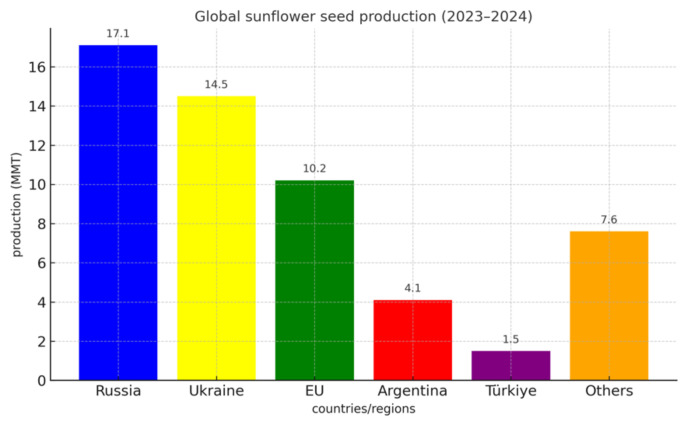
Global sunflower seed production (2023–2024).

Sunflower (*Helianthus annuus*) holds a prominent position among oilseed crops cultivated around the globe due to its adaptability, agronomic resilience, and utility across multiple sectors. Its suitability for diverse climatic zones—ranging from temperate to subtropical regions—has facilitated its widespread cultivation at both subsistence and industrial scales. During the 2023–2024 agricultural cycle, global sunflower seed production was reported at approximately 55 million metric tons (MMT), with Russia contributing the largest share (17.1 MMT), followed by Ukraine (14.5 MMT). The European Union accounted for 10.2 MMT, whereas Argentina and Türkiye produced 4.1 MMT and 1.5 MMT, respectively. The remaining production, totaling around 7.6 MMT, was distributed among several other producing regions^1^ (Figure 1).

The economic relevance of sunflower cultivation extends beyond its yield volume. Sunflower oil, widely consumed as a cooking medium, is recognized for its nutritional attributes and health-promoting properties, which include a favorable fatty acid profile and oxidative stability (Puttha et al., 2023). The residual sunflower meal obtained after oil extraction serves as a protein-rich feed component within the livestock and poultry sectors. Additionally, its therapeutic and cosmetic applications have been acknowledged in the pharmaceutical and skincare sectors, particularly due to its antiinflammatory and antioxidant activity. In recent developments, the utilization of sunflower oil in biodiesel production has further expanded its industrial footprint (Adeleke and Babalola, 2020). Taken together, these applications underscore the crop’s role not only in enhancing food security but also in supporting economic growth and industrial sustainability (Grompone, 2020).

However, sunflower production is increasingly being threatened by plant diseases that can severely reduce yield and affect crop quality. These diseases, caused by fungal, bacterial, or viral pathogens, can attack at various growth stages, often leading to defoliation, poor seed formation, and in severe cases, total crop loss (Bán et al., 2023). Downy Mildew (*Plasmopara halstedii*) and Rust (*Puccinia helianthi*) are two of the most common diseases and have been reported to reduce yields by 30%–90% in severe infestations (Bán et al., 2023; Hale, 2023). Others, such as Sclerotinia Stem Rot and Gray Mold, affect the structural integrity of the plant, while Bacterial Leaf Spot (*Pseudomonas syringae*) and Sunflower Mosaic Virus (SMV) play a significant role in limiting overall productivity (Gulya et al., 2019). If left unchecked, these diseases can not only cause direct yield loss but also increase management costs and disrupt the overall supply chain of sunflower-based products.

### 1.2. Rise of transfer learning in plant disease detection

Traditionally, diseases in sunflower crops have been detected through manual observation in the field based on the visual identification by the farmer of such symptoms as leaf discoloration, lesions, or wilting (Centorame et al., 2024). While this approach works to some extent for experienced farmers, it has clear limitations, being time-consuming, subjective, and often ineffective when symptoms are mild or in the early stages. Laboratory methods like molecular diagnostics and pathogen culturing provide better accuracy, but are expensive, slow, and unsuitable for large-scale or real-time monitoring (Centorame et al., 2024).

In response to disease outbreaks, chemical interventions—particularly applications of fungicides and pesticides—have been widely adopted by farmers as a primary management strategy (Centorame et al., 2024). While these methods offer short-term relief, their repeated use leads to increased production costs, environmental degradation, and the emergence of resistant pathogen strains, and their adverse effects on soil quality, microbial diversity, and ecological sustainability have been frequently reported. In this regard, there is a pressing need for early and accurate disease detection mechanisms that can serve as viable alternatives to chemical approaches (Dyussembayev et al., 2021).

Over the past decade, artificial intelligence (AI) has shown considerable promise across multiple domains, including finance (Gulzar et al., 2023a), medicine (Gulzar and Khan, 2022; Gulzar et al., 2025), and agriculture (Gulzar, 2025a, 2025b). AI technologies have been adopted in the agricultural sector to enhance precision farming, regulate irrigation practices, and automate operations such as pest detection and weed removal (Shaikh et al., 2022). Specifically, deep learning (DL) approaches—most notably, convolutional neural networks (CNNs) and more recently, vision transformers (ViTs)—have been employed for the diagnosis of plant diseases through the processing of high-resolution plant imagery, facilitating the identification of minute pathological features with commendable accuracy (Seelwal et al., 2024).

The development of such models from the ground up, however, typically necessitates access to extensive labeled datasets and substantial computational resources. These prerequisites often remain unmet in agricultural contexts, particularly for underrepresented crops such as sunflower (Xu et al., 2023; Centorame et al., 2024). In this regard, transfer learning (TL) can be considered a practical and efficient alternative. By leveraging pre-trained models originally optimized on large-scale datasets such as ImageNet, and subsequently fine-tuning them using domain-specific data (e.g., sunflower disease imagery), researchers have been able to reduce training overheads, improve model robustness, and address data scarcity issues (Zhao et al., 2024). TL has exhibited favorable generalization capabilities and has been especially beneficial in scenarios where annotated samples are limited. While promising results have been observed in crops such as tomato, maize, and potato, the use of TL in sunflower disease detection remains a relatively underexplored domain. Consequently, further investigation into model customization, data augmentation strategies, and real-world deployment frameworks is warranted to realize its full potential in operational environments (Amri et al., 2024).

### 1.3. Research gap, motivation, and objectives

Deep learning models have shown favorable results in plant disease classification tasks; however, their success largely depends on the availability of sufficient training data and model generalizability—two areas in which sunflower disease detection is lacking. The key challenges in this domain include:

- Limited labeled datasets: Most available plant disease datasets do not include sunflower images, and the few that exist are usually small and imbalanced.
- Domain shift: Pretrained models like ResNet, Inception, and ViT are trained using generic images, and their performance often declines when applied to real-world agricultural data, particularly under field conditions.
- Deployment challenges: High-end models are not always practical for farmers or agricultural workers in the field, and devices with limited computing capacity cannot run them efficiently.

Transfer learning can be considered a promising approach to overcoming these limitations, leveraging existing models and adjusting them for use in new tasks based on limited data. However, for TL to be fully effective in sunflower disease detection, more work is needed to customize architectures, improve accuracy in complex field scenarios, and simplify model deployment.

### 1.4. Review objectives

This review has the following objectives:

1. To summarize existing studies in which transfer learning has been adopted for sunflower disease detection.
2. To compare the performance of various TL-based architectures, including CNNs and ViTs.
3. To outline the key challenges currently limiting progress in this area.
4. To propose future directions for research, especially in the fields of dataset development, model optimization, and deployment strategies in the field.

## 2. Literature retrieval strategy and inclusion criteria

A structured literature search of the Scopus database was conducted to identify academically credible and peer-reviewed sources. Scopus was specifically chosen due to its wide indexing coverage of such major publishing houses as Elsevier, Springer, Wiley, IEEE, Taylor & Francis, MDPI, Hindawi, and Emerald, among others. Its citation tracking capabilities, metadata consistency, and inclusion of high-quality conference proceedings further justified its selection over other databases. Sources such as Google Scholar and ResearchGate were intentionally excluded due to their indexing inconsistencies and the presence of non-peer-reviewed and preprint materials, which fall outside the scope of this review.

The literature was searched using the following query, applied to the Title, Abstract, and Key words fields:

*TITLE (sunflower OR “Helianthus annuus” OR “Helianthus spp.” OR “H. annuus”) AND TITLE-ABS-KEY (disease) AND TITLE-ABS-KEY (“transfer learning” OR “deep learning” OR dl OR ai OR cnn OR “convolutional neural network”)*

This query was carefully constructed to capture works related specifically to sunflower disease and the use of AI-driven techniques, particularly transfer learning (TL) and deep learning (DL).

An initial total of 47 documents was retrieved, which included:

- 26 articles
- 19 conference papers
- One book chapter
- One data paper

To narrow the scope to include only relevant and technically sound literature, the 47 documents were screened based on the following inclusion criteria:

- Studies addressing sunflower disease detection, classification, or diagnosis using deep learning, AI, or specifically transfer learning techniques.
- Articles published in peer-reviewed journals or conferences indexed in Scopus.
- Studies written in English.

The following exclusion criteria were also applied:

- Articles not directly related to sunflower diseases or lacking AI- or TL-based methodologies.
- Papers written in languages other than English.
- Duplicate entries in different publications.
- Articles lacking experimental implementation, algorithmic discussion, or technical depth (e.g., opinion pieces, editorials, or general reviews unrelated to TL).

This screening process led to the exclusion of several documents. The remaining set of eligible studies formed the basis of this review and were used in the subsequent analysis and comparison of TL-based techniques for sunflower disease detection.

**Figure 2:**
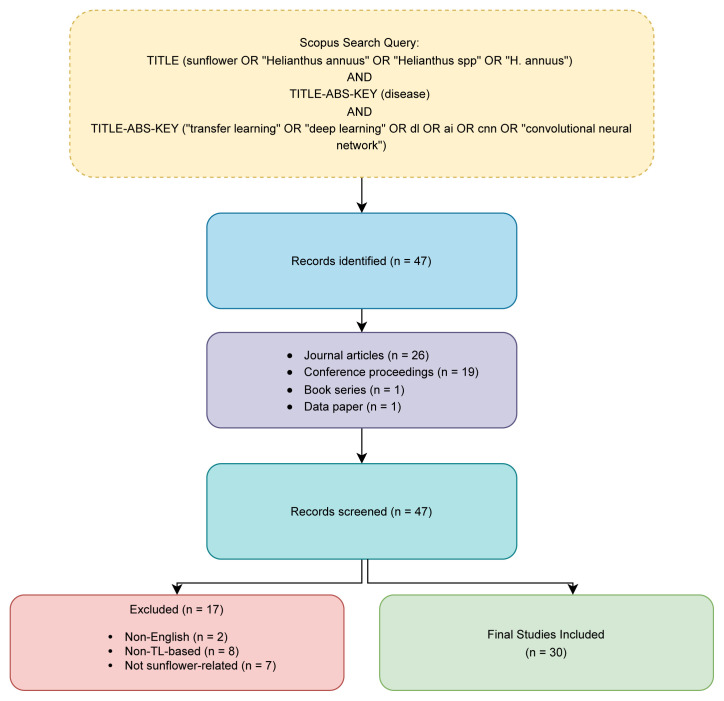
PRISMA flow diagram of study identification, screening, and inclusion.

This focused and selective approach was considered essential to ensure that the discussion remained rooted in peer-reviewed, technically grounded, and practically relevant research. Following the application of the stated criteria, a total of 30 studies were found suitable for detailed review and analysis, while 17 documents were excluded for such reasons as language constraints, lack of relevance to TL-based techniques, or irrelevance to sunflower-specific disease detection. A visual summary of the search strategy, screening stages, and inclusion criteria is presented in Figure 2.

## 3. Transfer learning techniques in sunflower disease detection

### 3.1. Common architectures and pretrained models used

A total of 30 studies were critically examined, revealing the predominant application of convolutional neural networks (CNNs) as the foundational architecture. Both standalone and hybrid implementations were observed, and classical architectures such as VGG-16, ResNet-50, InceptionV3, and DenseNet201 were frequently employed with transfer learning. Studies S1, S5, S10, S12, and S25 employed pretrained weights and fine-tuning techniques to adapt general-purpose feature extractors to the specific requirements of sunflower disease classification.

Notably, EfficientNetB3 (S12) and EfficientNetV2-Small (S27) outperformed several of the earlier models, delivering superior accuracy while maintaining lower computational overheads. Lightweight CNN variants such as MobileNetV3-Small, ShuffleNetV2, SqueezeNet, and MnasNet-A1 (S27) showed significant promise in resource-constrained settings, while TeenyNet (S22)—a custom lightweight architecture incorporating multi-frequency feature extraction and attention fusion—demonstrated high performance (98.94%), despite its 143 KB size, highlighting its suitability for mobile and embedded deployments.

Hybrid model configurations were adopted in numerous studies. The integration of CNNs with such classical classifiers as Random Forest (S21, S28, S30) and Support Vector Machines (S8, S13, S29) resulted in notable performance improvements over their nonhybrid counterparts. Federated learning was addressed in studies S14, S17, S18, and S20, wherein distributed client data were processed without centralized aggregation. These studies reported accuracy gains following federated averaging, with S17 documenting an increase from 88.76% to 95.72%.

Advanced configurations were also noted. Study S26 investigated diffusion models augmented with attention layers under a few-shot learning paradigm, while YOLOv5-based object detection frameworks were enhanced in S16 using integrated domain-specific optimization modules (IDMO). An ensemble learning approach using CNN and AdaBoost (S15) achieved improved disease severity classification in field images. The previously reported limitations in field performance in S4 were addressed in later studies, achieving an accuracy of 98.02%.

### 3.2. Dataset properties and disease class distribution

The datasets utilized in the studies varied significantly in terms of scale, disease class representation, and the applied acquisition method. Smaller datasets, often under 500 images (S1, S5, S11, S25, S29), were developed primarily from custom image scraping or lab setups. In contrast, larger datasets such as those in S9, S24, S28, and S30 were based on thousands of annotated images, and achieved broader disease class coverage and environmental diversity. The most frequently encountered disease categories included downy mildew, *Alternaria* leaf spot, rust, *Phoma* blight, gray mold, and *Verticillium* wilt. Some of the studies were broader in scope, incorporating disease severity levels (S15, S18, S20) or leveraging hyperspectral imaging for nonvisible infestation markers (S23).

Balanced datasets were reported in S9 and S24, in which careful curation contributed to improved classifier performance. The preprocessing pipelines generally included image resizing, contrast normalization, color enhancement, and augmentation (e.g., flipping, rotation). In S25, stratified k-fold cross-validation was employed to reduce sample bias and to validate statistical consistency across training iterations. The use of hyperspectral Vis–NIR imaging in integration with CNN and Extreme Learning Machine (ELM) in S23 facilitated the nondestructive early detection of *O. cumana* infestation and cultivar differentiation with an accuracy exceeding 95%.

The data sources ranged from open-access platforms such as Kaggle (S16), curated field imagery (S4, S9, S25), to synthetic scraping from online repositories (S1, S5). The presence of real-world noise, such as background clutter and varying lighting conditions, differed across datasets. Studies employing federated learning (S14, S17, S18, S20) simulated client-side heterogeneity for the realistic representation of geographically distributed agricultural environments.

### 3.3. Performance evaluation and comparative outcomes

Model performance was evaluated based on common classification metrics, namely accuracy, precision, recall, F1-score, and area under the curve (AUC). Among the reviewed implementations, EfficientNetV2-Small (S27) yielded the highest reported classification accuracy of 99.20% following transfer learning adaptation. The Hybrid CNN-RF architectures utilized in S28 and S30 achieved reported validation accuracies of 96.2% and 97.0%, respectively, confirming the efficacy of ensemble configurations.

In studies employing federated learning, metrics were reported using macro, micro, and weighted averages to capture multiclass and cross-client performance dynamics. In S17, a significant improvement was reported in the macro average, from 88.76% to 95.71%, across five simulated clients. Studies S18 and S20 demonstrated consistent behavior across imbalanced data distributions, indicating the resilience of the federated training protocol.

The application of feature fusion and multi-stage training pipelines (S13, S15, S16) led to notable performance enhancement. In particular, the IDMO-enhanced YOLOv5 framework adopted in S16 aided in the accurate detection of subtle disease features through effective segmentation. Disease severity staging was addressed in S15 and S25, with S25 reporting accuracies of 90.25% and 86.89% in *Alternaria* and powdery mildew classification using fine-tuned VGG models with stratified sampling.

Real-world applicability remains a key concern. While the majority of studies demonstrated high internal validation accuracy, their suitability for field conditions varied. Study S6 reported 89.15% accuracy based on real field images, although certain disease types, such as powdery mildew, posed classification challenges, although performance discrepancies in previously reported underperforming models, such as S4, were resolved through retraining and dataset augmentation.

Overall, findings favor the use of hybrid architectures, lightweight CNNs with efficient transfer learning, and federated learning schemes. When combined with comprehensive preprocessing and diverse datasets, these configurations showed the highest potential for scalable and deployable solutions in precision agriculture.

A comprehensive summary of the model configurations, datasets, disease classes, and outcomes of all 30 studies is provided in Table 1.

## 4. Key word cooccurrence analysis and conceptual trends

To gain a deeper understanding of the intellectual landscape and thematic directions in the reviewed studies, a cooccurrence key word analysis was conducted using VOSviewer. Author and index key words were extracted from all 30 selected studies, and following preprocessing (involving the unification of such related terms as “leaf disease” “sunflower” and “sunflower disease”), the key words were analyzed to identify prominent concepts, their relationships, and research clusters. A minimum key word occurrence threshold of 2 was applied, resulting in the identification of 40 high-frequency terms.

**Figure 3:**
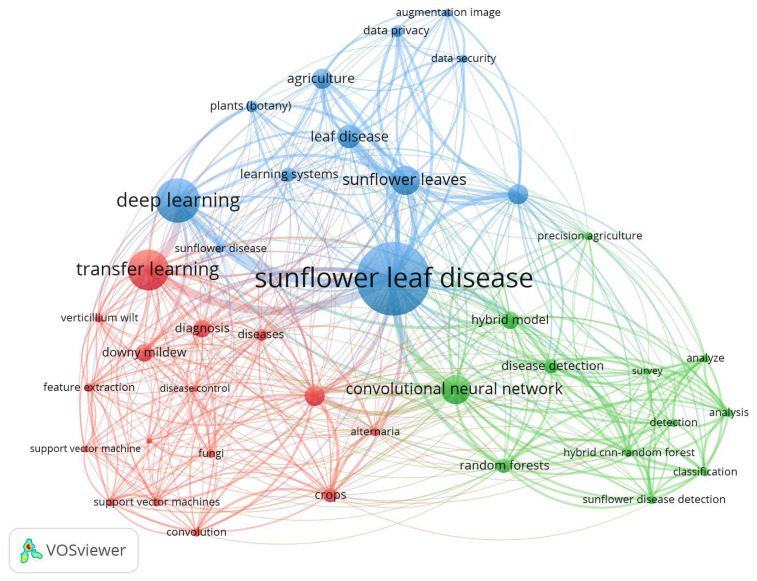
Network visualization of key word cooccurrence from 30 selected studies on sunflower disease detection using transfer learning

The interlinked structure of the key research themes are revealed in the network visualization (Figure 3), revealing five major clusters, each of which is represented by a unique color. The co-related areas identified include: (i) deep learning architectures and techniques, (ii) disease classification in sunflower crops, (iii) lightweight and hybrid models, (iv) image analysis and preprocessing methods, and (v) deployment contexts, including precision agriculture and federated learning. Notably, the cluster containing “transfer learning”, “CNN”, “feature extraction”, and “performance metrics” is at the core of the network, suggesting their centrality in most reviewed works.

**Figure 4:**
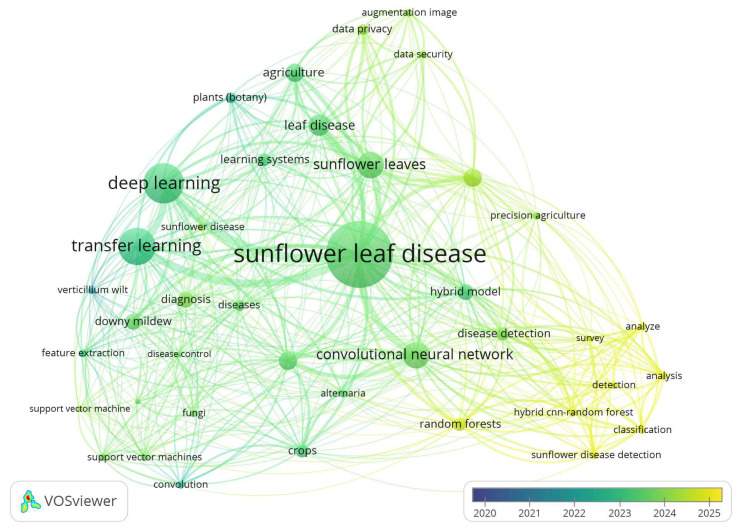
Overlay visualization showing temporal trends in key word usage from 2021 to 2025.

To complement the structural overview, an overlay visualization (Figure 4) was generated to map the temporal evolution of key word usage. Here, the node colors indicate the average year of key word occurrence, revealing the changes witnessed in research interests. In earlier studies (2021–2022), such general AI terms as “deep learning”, “CNN”, and “image classification” are prominent, while in more recent publications (2023–2025), themes such as “federated learning”, “lightweight CNN”, “data privacy”, “explainable AI”, and “severity analysis” are more common reflecting a growing focus on deployment feasibility, ethical AI, and robustness in real-world agricultural applications.

This trend is indicative of a natural progression of research maturity—from foundational model applications toward optimization, explainability, and scalability. Notably, key words such as “TeenyNet”, “diffusion models”, and “multi-frequency feature extraction” were noted only in the most recent studies, underscoring a shift toward novel architectures and embedded system compatibility.

The co-occurrence analysis revealed sunflower disease detection based on transfer learning to be a rapidly evolving area of research, progressing from initial feasibility demonstrations to more advanced solutions considering field conditions, dataset heterogeneity, and interpretability. All the above contribute to the thematic foundation upon which the challenges and future directions discussed in the subsequent sections are built.

## 5. Challenges and open research directions

Despite the notable progress in the use of transfer learning techniques for sunflower disease detection, several methodological and practical challenges remain unresolved. These challenges, revealed through the comparative analysis and identifiable from the key word trends, can be linked to the acquired data, model architecture, and the deployment and evaluation tasks, and continue to constrain scalability, accuracy, and real-world utility.

### 5.1. Dataset imbalance and diversity constraints

One recuring limitation that was notable in the reviewed studies was the lack of large, balanced, and environmentally diverse datasets. While some studies (e.g., S24, S28, S30) sought to mitigate these limitations through the use of curated or synthetically expanded datasets, many (S1, S5, S11) continued to rely on small-scale or unbalanced samples, often collected under laboratory conditions. Consequently, the generalizability of trained models to field scenarios—where disease manifestations are influenced by variable lighting, background clutter, and mixed infections—remained limited. This indicates a pressing need for large, annotated datasets obtained through multi-location field trials capturing a wide range of disease progression stages and environmental contexts.

### 5.2. Architectural adaptability and domain mismatch

While CNN-based models such as ResNet, VGG, and DenseNet have yielded strong baseline results, they were initially developed for generic image classification tasks, and their direct application to plant pathology data—especially sunflower leaf images with subtle disease signatures—has often led to suboptimal performance due to domain mismatch. Although several studies employed fine-tuning, feature fusion, or hybridization strategies (e.g., CNN-RF or CNN-SVM combinations), a more robust approach would involve the development of customized, agriculture-specific deep learning models that take leaf texture, vein patterning, and multi-class symptom overlaps into account with higher precision. Moreover, transformer-based models and attention-augmented architectures remain underexplored within this domain.

### 5.3. Federated learning scalability and edge deployability

Federated learning (FL) has emerged as a promising approach to decentralized training that removes the need for the transfer of sensitive data. While studies such as S14, S17, and S20 have reported encouraging performance gains using FL, especially under simulated client heterogeneity, the challenges associated with real-time synchronization, communication overheads, and robustness to data drift remain inadequately addressed. In addition, only a few models are compatible with edge devices or low-power computational environments. While lightweight networks such as TeenyNet (S22) and SqueezeNet variants (S27) have shown potential, further explorations are required to benchmark their performance under such field constraints as intermittent connectivity, hardware variability, and thermal thresholds.

### 5.4. Explainability, trust, and human-in-the-loop systems

The adoption of explainable AI (XAI) in sunflower disease detection has thus far been sporadic, with only a handful of studies (e.g., S11) investigating the use of interpretation tools such as LIME or saliency maps to validate the decision-making logic of their models. In agricultural settings, where user trust and interpretability are paramount for adoption, this remains a significant shortfall. Future studies would benefit from the inclusion of model interpretability as a core component, enabling agronomists and field workers to understand, verify, and contest predictions when needed. The integration of human-in-the-loop mechanisms, possibly through active learning frameworks, may further enhance system reliability and user confidence.

### 5.5. Handling multi-stage severity and mixed infections

Another notable gap is apparent in the limited capacity of existing models to accurately classify disease severity levels or detect overlapping infections. While S15 and S18 addressed severity quantification to some extent, most of the reviewed studies treated diseases as discrete classes, thereby overlooking the possible effects of intraclass variability when making treatment decisions. The development of models that can handle fine-grained classifications—such as for the identification of early, intermediate, and advanced stage infections—and that incorporate multilabel classification capabilities, will be essential for real-time agronomic decision support.

A consolidated view revealing the major gaps identified in the literature is presented in Table 2.

### 5.6. Interdisciplinary potential and cross-crop adaptability

Although the scope of this review is limited to sunflower, the challenges, architectures, and proposed solutions may be generalized to other broadleaf crops, such as soybean, maize, cotton, and tobacco. The classification of diseases in these crops often face similar constraints, including scarce labeled data, mixed disease types, and the need for deployment in resource-constrained environments. The findings synthesized in this review can thus inform the design of TL-based frameworks for a broader range of plant species, fostering knowledge transfer across domains and supporting the development of more universally applicable models.

## 6. Practical implications and real-world deployment considerations

The insights presented in this review, particularly those synthesized from the 30 peer-reviewed studies, offer significant implications for the practical deployment of transfer learning models in real-world sunflower disease detection scenarios. Although promising results have been reported across diverse architectures, datasets, and methodologies, deployment challenges remain a persistent barrier to field-level application. This section reflects on the applicability, feasibility, and limitations of the use of the reviewed approaches in operational agricultural environments, with specific emphasis on field readiness, computational constraints, model interpretability, and integration into existing agronomic workflows.

### 6.1. Field readiness and deployment suitability

A large proportion of the reviewed studies (e.g., S6, S9, S10, S24, S25) achieved high classification accuracy (>90%) under experimental or semi-controlled conditions. However, the real-world transferability of these models remains uncertain under such heterogeneous conditions as inconsistent lighting, background noise, occlusion by soil or overlapping leaves, and variations in the expression of disease symptoms. While data augmentation techniques have been employed to simulate variability, most studies fail to validate the results based on field-acquired datasets, limiting the generalizability of the findings.

Only a few of the studies (e.g., S4, S15, S22, S23) reported on the performance of the applied model in authentic field settings, revealing a decline in model precision under the effects of environmental noise and non-standard disease presentations, thus affecting classification confidence. The ability of TL models to operate reliably under such variable conditions must therefore be considered a critical determinant of field readiness.

### 6.2. Computational constraints and model efficiency

The limits on resources often encountered in rural farming environments—particularly in low- and middle-income countries—place practical limitations on the deployment of high-capacity DL models. Standard CNN architectures such as ResNet152, DenseNet201, and InceptionV3, though effective, may be incompatible with low-power devices due to their memory and computational demands.

In such contexts, lightweight architectures (e.g., MobileNetV3-Small, ShuffleNetV2, TeenyNet) have shown significant promise. TeenyNet (S22), which is just 143 KB in size, achieved 98.94% accuracy under natural light conditions, supporting its use in mobile and embedded systems. Similarly, hybrid implementations combining CNN feature extractors with classical machine learning classifiers (e.g., SVM, RF), reported in studies such as S8, S13, S21, and S28 yielded competitive accuracy with reduced complexity. These findings suggest that future real-world systems may benefit from prioritizing compactness and resource-efficiency, making them suitable for use in edge deployment scenarios.

### 6.3. Federated learning and data privacy considerations

Federated learning has recently emerged as a viable approach to collaborative model training without the need for centralized data aggregation. Studies S14, S17, S18, and S20 effectively demonstrate the feasibility of FL in agricultural contexts, reporting accuracy gains of up to 95.72% while preserving data privacy across client nodes.

In operational terms, FL addresses a key bottleneck in agricultural AI, namely, the reluctance or logistical challenges associated with the aggregation of sensitive farm data across geographies. FL facilitates large-scale training under privacy-preserving frameworks by allowing individual farms or regions to contribute to the improvement of the model without disclosing local data. The practical deployment of FL, however, requires stable connectivity, synchronization mechanisms, and cross-device coordination, which are not yet commonly available in many sunflower-growing regions. To overcome this limitation, further research is needed to develop FL protocols that can support their use in asynchronous, low-bandwidth environments with intermittent power supplies.

### 6.4. Interpretability and farmer trust

In addition to accuracy and efficiency, model transparency remains a critical factor influencing user trust and adoption. Farmers and agronomists are unlikely to rely on black-box models unless decisions can be traced, interpreted, and justified. Techniques such as class activation mapping, saliency map, and Local Interpretable Model-agnostic Explanation (LIME) techniques explored in S4, S11, and S12, can make a positive contribution to the field by providing visual explanations of the offered predictions.

That said, the adoption of explainable AI (XAI) in the reviewed studies remains limited, with only a minority of works explicitly addressing interpretability, and even fewer providing actionable visual outputs suitable for non-technical users. To ensure meaningful field integration, future systems must be embedded with interpretability features that can be understood by local stakeholders, possibly through mobile interfaces that highlight affected regions, annotate disease types, and recommend specific responses.

### 6.5. Integration into agricultural ecosystems

The deployment of TL-based disease detection models should not be viewed as an isolated technological task, but rather as part of a broader ecosystem that includes agronomic advisory systems, pest management protocols, and crop monitoring platforms. Seamless integration with agricultural management systems (AMS), remote sensing data, and decision-support tools is essential for the derivation of practical value.

Models optimized for standalone image classification offer limited utility unless coupled with temporal monitoring, geotagging, or actionable insights. For example, integration with drone-based imaging (S3, S16) and hyperspectral systems (S23) can enhance scalability and detection accuracy over large fields. Similarly, model predictions should ideally feed into extension service platforms or agronomic dashboards to support timely interventions by farmers or agricultural officials.

### 6.6. Summary of deployment considerations

Table 3 provides a summary of deployment-relevant attributes observed across the reviewed studies, consolidating the above discussions. Key factors such as field validation, model size, training complexity, and interpretability have been highlighted to guide researchers and practitioners toward the most appropriate models for real-world applications.

## 7. Future research directions

Although significant progress has been made in the utilization of transfer learning for the detection of sunflower diseases, there remain several research gaps and newly emerging themes that warrant further investigation. The following future directions are proposed to enhance the robustness, scalability, and practical utility of models in diverse agricultural scenarios.

### 7.1. Development of multimodal and context-specific models

Most studies to date have relied solely on RGB image inputs for disease detection. While effective under controlled conditions, this approach remains limited in its capacity to capture the physiological and environmental context influencing disease manifestation. Future studies should prioritize the development of multimodal frameworks that combine RGB imagery with hyperspectral, thermal, or biochemical data. Studies such as S23 have shown how the integration of Vis–NIR hyperspectral data can enhance early disease detection and cultivar differentiation. Incorporating such contextual metadata as geolocation, weather parameters, and growth stage information could contribute further to diagnostic precision.

### 7.2. Adaptive and domain-specific architectures

It is apparent that the existing, predominantly CNN-based TL architectures were developed for general-purpose image classification, and their direct application to agronomic datasets introduces limitations related to domain mismatch and class overlap. As such, there remains a critical need for agriculture-specific models that consider leaf morphology, venation patterns, and symptom localization. Architectures incorporating deformable convolutions, attention-based saliency mechanisms, or hierarchical feature extraction modules should be further explored. Additionally, transformer-based models and few-shot learning strategies, such as those evaluated in S26, warrant broader investigation in future studies.

### 7.3. Advanced handling of disease severity and multilabel classification

A predominant focus on single-label classification is apparent in the reviewed studies, with limited attention paid to intraclass variability or coinfections. Since sunflower diseases often present as a spectrum of severity or occur simultaneously with overlapping symptoms, future studies should investigate the development of multilabel classification frameworks that can reveal both the presence and severity of multiple diseases. Incorporating ordinal classification techniques and severity regression pipelines could enhance decision-making precision in agronomic practice.

### 7.4. Realistic benchmarking and public dataset initiatives

The scarcity of open-source, balanced, and field-acquired sunflower disease datasets continues to limit the reproducibility and comparative evaluation of studies in the field. To address this, future initiatives should contribute to the creation of open-access benchmark datasets that reflect the real-world variability in environmental conditions, disease severity, and image acquisition. Such datasets should include metadata annotations, standard train-test splits, and multi-site samples, thus facilitating robust benchmarking and cross-study comparability.

### 7.5. Embedded systems and low-cost deployment solutions

The effective deployment of TL models in field conditions and the accuracy of the findings depend largely on the compatibility of the hardware and the stability of the system. Future studies should prioritize the optimization of models for real-time use on edge devices such as smartphones, drones, and agricultural robots. This will involve a deeper exploration of pruning, quantization, and knowledge distillation techniques with the goal of reducing model complexity without sacrificing accuracy. While studies such as S22 and S27 have made progress in this regard, there remains a lack of studies in which standardized performance is evaluated under actual deployment scenarios.

### 7.6. Human-in-the-loop and farmer-centric interfaces

While automation remains a core objective, the integration of human expertise into the decision-making pipeline should not be abandoned. Active learning frameworks that allow iterative model improvement based on user feedback can enhance system reliability and user trust. Furthermore, the design of intuitive user interfaces, tailored to suit the literacy and technology familiarity levels of end-users—particularly smallholder farmers—must be considered a priority in future deployment studies. Mobile applications that provide interpretive feedback, visual cues, and agronomic recommendations based on model predictions can bridge the gap between AI outputs and actionable practice.

### 7.7. Cross-crop transferability and regional adaptation

Transfer learning offers unique advantages in facilitating cross-crop and cross-regional knowledge transfer. Models that are fine-tuned for sunflower diseases may serve as a foundation for the creation of models with similar foliar disease patterns, such as soybean, maize, and cotton. The development of generalizable frameworks and domain adaptation protocols that enable rapid tuning for specific crop types and agroclimatic zones will enhance the scalability of AI solutions in precision agriculture. Comparative studies examining the transferability of TL models across species and regions should be conducted to establish guidelines for model adaptation.

Table 4 presents a summary of the proposed strategic directions, outlining the key future research priorities and core areas for study, including data acquisition, model design, deployment readiness, interpretability, and domain adaptability.

## 8. Conclusion

The critical examination the role of transfer learning in advancing sunflower disease detection presented here is based on an analysis of 30 peer-reviewed studies published over the last 5 years. The investigation of the model architectures, datasets, performance metrics, and deployment settings has revealed several key insights and persistent challenges. Despite notable improvements in classification accuracy and computational efficiency, there are several core limitations that remain unresolved related to dataset diversity, domain adaptability, explainability, and real-world deployment readiness.

The analysis reveals that most existing TL applications have been validated only in experimental settings, while their performance in heterogeneous field conditions remains untested. Furthermore, despite the promising results achieved with hybrid and lightweight models, their integration into edge-based agricultural workflows remains underexplored. Similarly, the sporadic adoption of explainable AI techniques and the absence of human-in-the-loop mechanisms have constrained user trust and interpretability—two critical factors that would support widespread adoption among end-users such as agronomists and farmers.

From a methodological standpoint, the continued reliance on generic CNN architectures, often repurposed without domain-specific adaptations, underscores the need for customized models attuned to the unique visual and contextual features of plant pathology. Furthermore, the challenges associated with severity grading, co-infection detection, and federated learning deployment suggest that there are several technical and practical barriers that are still to be overcome before TL-based systems can be scaled reliably in operational settings.

Importantly, this review highlights the potential of TL models to undertake similar diagnostic tasks in other broadleaf crops facing analogous disease detection challenges. The future of this research domain will depend not only on algorithmic refinement, but also on the collaborative development of benchmark datasets, robust evaluation protocols, and application frameworks that are aligned with the sociotechnical realities of agriculture.

In conclusion, although transfer learning has proven to be a powerful tool for the early and accurate detection of plant disease, its full potential for sunflower pathology has yet to be realized. Addressing the identified gaps through targeted research and cross-sector collaboration will be essential for the transformation of these models from academic prototypes into scalable, field-ready tools capable of supporting sustainable and data-driven crop health management.

## Figures and Tables

**Table 1:** Overview of deep learning and transfer learning configurations used for sunflower leaf disease classification.

Study no.	Year	Models used	Dataset source	Disease classes	TL used	Accuracy /outcome
S1 (Sirohi and Malik, 2021)	2021	VGG-16 + MobileNet	Google Images (Custom)	Alternaria, Downy mildew, Phoma, Verticillium	Yes	89.20%
S2 (Dawod and Dobre, 2021)	2021	ResNet152	Improved dataset	4 foliar diseases	Yes	Not stated
S3 (Chen et al., 2021)	2021	Yolov4 + MobileNetV1–3	Inner Mongolia	Verticillium, powdery mildew, rust	Yes	84%
S4 (Dawod and Dobre, 2022a)	2022	ResNet	Field Dataset	4 foliar diseases	Yes	98.02%
S5 (Malik et al., 2022)	2022	VGG-16 + MobileNet (stacked)	Google Images (329 images)	Alternaria, Phoma, downy mildew, Verticillium, healthy	Yes	89.2%
S6 (Dawod and Dobre, 2022b)	2022	Faster R-CNN, Mask R-CNN + ResNet50/152	Field Dataset (858 images)	Alternaria, rust, powdery mildew, healthy	Yes	89.15% (ResNet152/DS1-V2)
S7 (Sara et al., 2022)	2022	Field Dataset (467 Images)	BARI Gazipur, Bangladesh	Downy mildew, gray mold, leaf scars, healthy	No	Dataset only
S8 (Banerjee et al., 2023)	2023	CNN + SVM	Not explicitly stated	Multiple diseases	No	Accuracy: 83.59%, F1: 83.45%
S9 (Sohel et al., 2023)	2023	InceptionV3, VGG19, MobileNetV2, ResNet152V2, DenseNet201	Bangladesh field dataset	Gray mold, downy mildew, leaf scars, healthy	Yes	DenseNet201: 98.7%
S10 (Sathi et al., 2023)	2023	ResNet50, InceptionV3, 2 other DL models	Custom dataset (1428 images)	Leaf scars, gray mold, fresh leaf, downy mildew	Yes	ResNet50: 97.88%; InceptionV3: lowest, 78.16%
S11 (Ghosh et al., 2023)	2023	VGG19 + CNN (Hybrid)	BARI Gazipur, Bangladesh	Downy mildew, gray mold, leaf scars, healthy	Yes	Proposed Model (VGG19 + CNN) 93.0%
S12 (Gulzar et al., 2023b)	2023	AlexNet, VGG16, InceptionV3, MobileNetV3, EfficientNetB3	Bangladesh field dataset	Gray mold, downy mildew, leaf scars, healthy	Yes	AlexNet 86.4%, VGG16 96.5%, InceptionV3 95.4%, MobilenetV3 96.9%, EfficientNetB3 97.6%
S13 (Rana et al., 2023)	2023	CNN (custom) + SVM (hybrid)	Custom dataset	Alternaria, downy mildew, Phoma, Verticillium	Yes	CNN-SVM model showed promising performance; better than traditional CNNs
S14 (Sharma et al., 2024)	2024	CNN + Federated Learning	Distributed/local	Different types of downy mildew scales	No	Accuracy improved from 82.95% to 94.88% using federated averaging techniques
S15 (Banerjee et al., 2024)	2024	CNN + AdaBoost	3,300 field images	Downy mildew (4 severity levels)	No	98.16% accuracy; hybrid model outperformed standalone CNN and AdaBoost
S16 (Thilagavathi and Arockiam, 2024)	2024	Enhanced YOLOv5 + IDMO	Kaggle dataset	Alternaria leaf spot, Verticillium wilt	NO	~95% accuracy; improved detection of subtle disease patterns via segmentation
S17 (Joshi et al., 2024)	2024	Federated CNN (5 clients)	Not specified	Common sunflower leaf diseases	Not Mentioned	Accuracy improved from 88.76% to 95.72% using Federated Averaging (xc_1 to xc_5)
S18 (Singh et al., 2024)	2024	CNN + Federated Learning	Client-specific data	4 disease severity levels	Not Specified	Macro avg: 87–90.35%, Micro avg: 90.41–94.83%, Weighted avg: 90.41–94.85%
S19 (Rani et al., 2024)	2024	Custom 4-layer CNN	Not mentioned	Downy mildew, rust, mild mildew, Alternaria, Septoria, Sclerotinia, bacterial, Verticillium, Fusarium, and cercosporin leaf	No	Macro avg: 92.19%, Micro avg: 92.20%, Weighted avg: 92.21%, Mean Accuracy: 84.11%
S20 (Suryavanshi et al., 2024)	2024	CNN + Federated Learning	Distributed clients (5 disease classes)	5 sunflower diseases	No	Macro: 85.31–91.85%, Weighted: 85.32–91.86%, Micro: comparable high performance
S21 (Yashu and Singh, 2024)	2024	CNN + Random Forest	4,356 web-sourced images	7 diseases: downy mildew, rust, Verticillium wilt, Phoma black stem, Alternaria leaf spot, Sclerotinia stem rot, Phomopsis stem canker	No	81.54% overall accuracy; F1-scores high for Phomopsis Stem Canker & Sclerotinia Stem Rot
S22 (Zhong and Tong, 2024)	2024	TeenyNet (lightweight CNN)	Sunflower leaves under natural light	Multiple (unspecified)	No	98.94% accuracy; model size only 143 KB; excels in edge and texture extraction
S23 (Li et al., 2024)	2024	ELM, CNN	Vis–NIR HSI + biochemical markers	O. cumana infestation; cultivar types	No	95.83% (infestation) and up to 97.92% (variety detection); non-destructive, early detection
S24 (Rajora et al., 2024)	2024	Deep learning model	9,695 curated images	Multiple (incl. blight)	No	Per-class accuracy: 96.65%–98.75%; overall precision: ~96.75%, blight: 99%
S25 (Sarode and Deshpande, 2025)	2025	VGG16, VGG19 (TL + FT)	Farms in Marathwada, India (Rabi season)	Alternaria, powdery mildew (3 stages + healthy)	Yes	VGG16: 90.25%, VGG19: 86.89%; small CI (~3–4%) at 95% confidence level
S26 (Zhou et al., 2025)	2025	Few-shot learning + Diffusion Models + Attention	Field Dataset	Brown spot, wilt, rust, black spot, downy mildew	Yes	Precision: 0.94, Recall: 0.92, Accuracy: 0.93, mAP@75: 0.92
S27 (Zhang and Wu, 2025)	2025	SqueezeNet, ShuffleNetV2, MnasNet-A1, MobileNetV3-Small, EfficientNetV2-Small	Custom (1,892 images)	Downy mildew, gray mold, leaf scar, fresh leaf	Yes	EfficientNetV2-Small achieved 99.20% accuracy after TL; all models improved
S28 (Mehta and Aneja, 2025a)	2025	CNN + RF (Hybrid), CNN, RF	Custom (10,000 images)	Downy mildew, rust, leaf blight, Alternaria leaf spot, healthy	No	CNN-RF hybrid achieved 96.2% accuracy, outperforming CNN (93.5%) and RF (85.1%)
S29 (More et al., 2025)	2025	ResNet-50 + SVM, RF, KNN, NB	Custom (466 images)	Downy mildew, fresh, gray mold, leaf scars	Yes	Best model (ResNet-50 + SVM) achieved 90% accuracy using CNN feature fusion
S30 (Mehta and Aneja, 2025b)	2025	CNN + Random Forest	Custom (5,000 images)	Twig & fruit blights, mildews, rusts, leaf spots, mosaic virus	No	Validation accuracy: 97%, AUC: 0.98, Precision/Recall/F1: ~0.96

**Table 2:** Summary of key research gaps and technical challenges in transfer learning for sunflower disease detection.

Category	Identified gap/limitation	Implication
**Dataset quality**	Small, imbalanced, or non-representative datasets	Low generalization in real-world agricultural settings
**Model adaptability**	Use of generic architectures not optimized for sunflower leaf characteristics	Domain mismatch, reduced accuracy for subtle or early symptoms
**Federated learning**	Limited testing under real-world client heterogeneity	Scalability and consistency in decentralized setups are affected
**Interpretability**	Lack of consistent application of explainable AI tools	Reduced user trust and model transparency
**Edge deployability**	Incompatibility with low-power or mobile devices	Constraints on use by farmers and field technicians
**Severity & mixed Infections**	Poor handling of disease stages or co-occurrence	Hinders targeted treatment and early intervention strategies

**Table 3:** Comparative summary of real-world deployment suitability across selected studies.

Study	Model type	Lightweight/hybrid	Field validated	Interpretability Used	Suitable for low-power devices
S4	ResNet	No	Yes	Yes	Moderate
S10	ResNet50	No	Yes	No	No
S13	CNN + SVM	Yes	No	No	Yes
S14	CNN + FL	Yes	Simulated	No	Yes
S15	CNN + AdaBoost	Yes	Yes	No	Yes
S17	Federated CNN	Yes	Simulated	No	Yes
S22	TeenyNet	Yes	Yes	No	Yes
S23	CNN + ELM	Yes	Yes	Partial	Yes
S27	EfficientNetV2-S	Yes	No	No	Yes
S28	CNN + RF	Yes	Yes	No	Yes

**Table 4:** Strategic priorities for future research in sunflower disease detection using TL.

Focus area	Recommended direction	Expected benefit
Data acquisition	Multimodal, annotated, field-validated datasets	Enhanced model generalization and benchmarking
Model development	Domain-specific CNNs, transformers, and severity regression pipelines	Improved performance for complex disease cases
Deployment	Edge-compatible, low-power models with feedback in real time	Broader field applicability
Interpretability	Integrated XAI visualizations and user-centric design	Increased trust and user engagement
Cross-domain Learning	Cross-crop and cross-region adaptation frameworks	Scalability and reduced retraining cost
Human interaction	Active learning with agronomist feedback loops	Continual model refinement and validation

## Footnotes

1 Major producer countries of sunflower seed (2023/2024) [Online] Website: https://www.statista.com/statistics/263928/production-of-sunflower-seed-since-2000-by-major-countries/# [Accessed February 24, 2025]
